# Delivery of Therapeutic miRNA via Plasma-Polymerised Nanoparticles Rescues Diabetes-Impaired Endothelial Function

**DOI:** 10.3390/nano13162360

**Published:** 2023-08-18

**Authors:** Yuen Ting Lam, Bob S. L. Lee, Juichien Hung, Praveesuda Michael, Miguel Santos, Richard P. Tan, Renjing Liu, Steven G. Wise

**Affiliations:** 1 Chronic Diseases Theme, School of Medical Science, University of Sydney, Sydney 2006, Australia; 2 Charles Perkins Centre, University of Sydney, Sydney 2006, Australia; 3 Victor Chang Cardiac Research Institute, Darlinghurst 2010, Australia; 4 St. Vincent’s Clinical School, University of New South Wales, Darlinghurst 2010, Australia; 5 School of Clinical Medicine, Faculty of Medicine and Health, University of New South Wales, Sydney 2050, Australia

**Keywords:** nanoparticles, miRNAs, endothelial, angiogenesis, diabetes, ischemia

## Abstract

MicroRNAs (miRNAs) are increasingly recognised as key regulators of the development and progression of many diseases due to their ability to modulate gene expression post-translationally. While this makes them an attractive therapeutic target, clinical application of miRNA therapy remains at an early stage and in part is limited by the lack of effective delivery modalities. Here, we determined the feasibility of delivering miRNA using a new class of plasma-polymerised nanoparticles (PPNs), which we have recently isolated and characterised. We showed that PPN-miRNAs have no significant effect on endothelial cell viability in vitro in either normal media or in the presence of high-glucose conditions. Delivery of a miRNA inhibitor targeting miR-503 suppressed glucose-induced miR-503 upregulation and restored the downstream mRNA expression of *CCNE1* and *CDC25a* in endothelial cells. Subsequently, PPN delivery of miR-503 inhibitors enhanced endothelial angiogenesis, including tubulogenesis and migration, in culture conditions that mimic diabetic ischemia. An intramuscular injection of a PPN-miR-503 inhibitor promoted blood-perfusion recovery in the hindlimb of diabetic mice following surgically induced ischemia, linked with an increase in new blood vessel formation. Together, this study demonstrates the effective use of PPN to deliver therapeutic miRNAs in the context of diabetes.

## 1. Introduction

MicroRNAs (miRNAs) are evolutionarily conserved small, non-coding RNAs that post-translationally inhibit or degrade target mRNAs [1]. miRNAs are estimated to regulate the expression of up to 60% of all protein-coding genes [2], making them essential players in various cellular processes, including development, differentiation, proliferation, and apoptosis. By fine-tuning gene expression, miRNAs ensure proper spatial and temporal gene-expression patterns that are crucial for normal physiological function. There is increasing evidence supporting the central role of miRNAs in the aetiology and progression of many diseases, including cancer [3,4], cardiovascular disease [5,6,7], diabetes [8,9,10], and neurodegenerative disorders [11,12,13]. Accordingly, miRNAs have emerged as promising therapeutic targets, primarily in the form of miRNA mimics or inhibitors, for the development of novel treatments for a wide range of diseases. miRNA inhibitors, also known as antimiRs, are single-stranded RNA molecules that bind to endogenous miRNAs and prevent them from interacting with target mRNAs. By blocking the activity of miRNAs, inhibitors can increase the expression of target genes and restore normal cellular function.

In diabetes, high blood glucose impairs vascular regeneration by interfering with angiogenesis. Diabetic patients have increased risks of cardiovascular disease and are more likely to develop vascular complications. Furthermore, diabetic individuals who experienced a delay in wound healing may be subject to lower-limb amputation in severe cases due to tissue death caused by ischemia. Many miRNAs have been identified as angiogenic mediators. These miRNAs positively or negatively modulate the function of endothelial cells (ECs), which line the inner layer of blood vessels and are the key contributors to blood vessel regeneration. Among these angiogenic miRNAs, some are explicitly associated with diabetes. For instance, miR-503, a member of the miR-16 family [14], is increased in the circulating blood and limb muscle tissues of diabetic patients who undergo lower-limb amputation [15]. Higher levels of miR-503 have also been found in diabetic patients with ischemic stroke, such that miR-503 may serve as an indicator of stroke severity [16]. Despite the identification of promising miRNA therapeutic targets, clinical translation of miRNA-based therapy remains at an early stage with numerous difficulties and challenges yet to be solved. 

One of the main limitations to the application of therapeutic miRNAs is delivery. Similar to other nucleic acids, negatively charged miRNAs are poorly taken up by cells due to limited passive diffusion across the cellular membrane. miRNAs are also easily degraded by nucleases in the bloodstream and in cells [17], can have non-specific effects on downstream tissue if not appropriately targeted, and in some cases, can have undesirable immunogenicity and toxicity effects. In recent decades, nanoparticle technology has become a rapidly growing area in the field of therapeutic interventions that promises to address some of these challenges. A wide variety of delivery platforms have been developed, focusing on optimising the design of nanoparticles to improve the safety and efficiency for miRNA delivery [18]. For example, lipid-based nanoparticles (LNPs), which encapsulate miRNA cargo in its core, protecting it from degradation and improving both targeting and delivery efficiency [19]. LNPs can also be modified with cell-penetrating peptides or targeting moieties to increase their specificity to target cells [20]. Mixed blends of synthetic or natural polymeric nanoparticles have also been described with improved biodegradability and biocompatibility to reduce potential cytotoxicity [21]. Inorganic nanoparticles, such as gold nanoparticles, conjugated with miRNA and targeting moieties have also been shown to increase delivery specificity and efficiency, though the underlying material brings some toxicity concerns [22].

Recently, we have developed a new class of nanoparticles that are synthesised by the process of plasma polymerisation [23]. Carbon-based plasma-polymerised nanoparticles (PPNs) are well tolerated by various types of cells with negligible cytotoxicity in vitro and in vivo, even at extremely high doses [24]. Functionalisation of PPNs with bioactive molecules is readily achieved in a one-step incubation in aqueous solution without the need of chemical linkers [23]. Furthermore, we have shown that PPNs were effective agents for the dual delivery of siRNA and anti-cancer drugs, paclitaxel, suppressing tumour growth in a mouse xenotransplant model of breast cancer [25]. In this study, we aim to investigate the therapeutic potential of PPNs in delivering miRNAs for improving endothelial function in the context of diabetes. We evaluated the cellular uptake and cytotoxicity of miRNA-functionalised PPNs and examined PPN-miRNA-mediated modulation of a target miRNA, miR-503, and its downstream mRNA expression. Furthermore, we assessed the effects of PPN delivery of miRNA on endothelial angiogenic function under diabetes-like culture conditions in vitro and a diabetic mouse model of hindlimb ischemia. Our study highlights the potential of using PPNs as a vehicle for the therapeutic delivery of miRNAs in vitro and in vivo.

## 2. Materials and Methods

### 2.1. PPN Synthesis, PPN-miRNA Conjugation and Characterisation

Plasma polymerisation nanoparticles (PPNs) were synthesised in a plasma reactor as previously described [23,25]. Synthesised PPNs were stored at room temperature and reconstituted in nuclease-free water prior t0 use. PPNs (10^9^ particles/mL) were conjugated with a miR-503 inhibitor oligonucleotide (Qiagen, Cat #339120Y104100899, Hilden, Germany), scrambled miRNA (Qiagen, Cat #339125Y100199006), or fluorescently labelled negative control miRNA (Integrated DNA Technologies, NC5 negative control, Coralville, IA, USA) in nuclease-free water for 1 h on ice. The amount of miRNA conjugated to PPNs is dependent on the ratio between PPN surface area and the molecular weight of miRNAs [23]. In this study, 1.6 µg (200 pmol) miRNA was conjugated to 10^9^ PPNs/mL (Supplementary Table S1). The hydrodynamic size distribution, concentration, and zeta potential of PPN were measured using a NanoSight NS300 laser-light-scattering system and a Zetasizer Nano ZS (Malvern Instruments, Herrenberg, Germany), as previously described [23]. 

### 2.2. In Vitro PPN-miRNA Binding Efficiency and miRNA Release

The binding efficiency of PPNs to a fluorescently labelled negative control miRNA (NC-Alexa647, 1.6 µg miRNA/10^9^ particles/mL) was determined by measuring free, unbound miRNA in an aqueous solution with a TECAN Infinite M1000 microplate reader [25]. The fluorescence intensities of serially diluted NC-Alex647 working stocks were measured with a TECAN Infinite M1000 microplate reader prior to PPN conjugation. After the conjugation, PPN-miRNAs were centrifugated at 21,000× *g* for 5 min to pellet the PPN-miRNA complex. The fluorescence intensity of unbound miRNA was measured in the supernatant at EX/EM: 650/665–671 nm. In the release study, the PPN-miRNA complex was washed after conjugation and resuspended in nuclease-free water or sodium citrate buffer (pH 7.2, to stimulate physiological, cytosolic pH) with or without foetal bovine serum (FBS). At pre-determined time points (0, 5, 10, 30, 60, 90, and 120 min), samples were centrifugated at 21,000× *g* for 5 min. The fluorescence signal from collected supernatants were measured by a TECAN microplate reader.

### 2.3. Cellular Uptake of PPN-miRNA and Its Effect on Viability

Human umbilical vein endothelial cells (HUVECs) were seeded on an 8-well chamber slide (40,000 cells/well) and treated with PPN-NC-Alex647 after 24 h seeding (1.6 µg miRNA/10^9^ particles/mL). Fluorescence images were taken at 2, 4, and 24 h. After 24 h of PPN-miRNA uptake, cells were fixed with 4% paraformaldehyde and stained with DAPI and F-actin (ActinRed555 Ready Probe Reagent, Invitrogen, Cat #37112, Waltham, MA, USA). Confocal images were taken with a Nikon AR-1 microscope. Flow cytometry analysis of the percentage of fluorescently positive cells within the population was performed with a BD FACSVerse flow cytometer. To examine the effects of PPN and PPN-miRNA on endothelial cell viability, HUVECs were treated with PPN controls or a PPN-scrambled miRNA complex. The concentrations of PPNs and PPN-scrambled miRNA (1.6 µg miRNA/10^9^ particles/mL) were serially diluted. A final concentration of PPN and the PPN-miRNA complex range from 10^3^ to 10^8^ particles/0.32 cm^2^ carrying 0.002–200 pmol (Supplementary Table S1). HUVECs were grown in a 37 °C incubator with 5% CO_2_ for 3 days. Cell viability was examined using Alamar Blue assay. Media were removed and replaced with fresh media containing 10% Alamar Blue reagent. After 2 h of incubation, fluorescence intensity of the Alamar Blue reagent (EX/EM: 530–560/590 nm) was measured using a TECAN Infinite M1000 microplate reader. Relative cell viability of PPN- and PPN-miRNA-treated cells was calculated and normalised against untreated HUVECs. 

### 2.4. miR-503 and mRNA Quantification

To determine the effect of PPN delivery of the miR-503 inhibitor on HUVEC expression of miR-503, cells were treated with the PPN-miR-503 inhibitor complex at 3 different concentrations: 0.016 µg miRNA/10^9^ PPNs; 0.16 µg miRNA/10^9^ PPNs; or 1.6 µg miRNA/10^9^ PPNs (Supplementary Table S2). After 24 h treatment, total RNA from HUVEC culture was extracted using mirVana PARIS RNA and a native protein purification kit according to the manufacturer’s protocol. Real-time quantification of miRNA was performed with the miCURY LNA microRNA PCR system (Qiagen, Hilden, Germany). miR-503 expression was normalised to U6 small nucleolar RNA (snRU6) using the comparative Ct method. Primer sequences are provided by manufacturer; the accession numbers are Hsa-miR-503: MIMAT0002874 and U6snRNA: X59362. For mRNA analysis, total RNA was extracted using TRIzol. cDNA was synthesised from 1 µg of total RNA and was amplified by real-time polymerase chain reaction (qPCR). Primer sequences included the following: *CDC25a*, forward: 5′-TAAGACCTGTATCTCGTGGCTG-3′, reverse: 5′-CCCTGGTTCACTGCTATCTCT-3′; *CCNE1*, forward: 5′-GAGCCAGCCTTGGGACAATAA-3′, reverse: 5′-GCACGTTGAGTTTGGG-TAAACC-3′, 18S ribosomal RNA, forward: GTAACCCGTTGAACCCCATT, reverse: CCATCCAATCGGTAGTAGGG. Each reaction was performed in triplicate. Expression levels of *CCNE1* and *CDC25a* were normalised with endogenous control 18S ribosomal RNA. Quantification was performed by 2^∆∆Ct^ method. Data were expressed as fold change. 

### 2.5. In Vitro Angiogenic Function: Tubulogenesis and Migration

Endothelial tubule formation was assessed in vitro using growth-factor-reduced Matrigel (Corning, Corning, NY, USA). Matrigel was stored at −20 °C until use. Thawed Matrigel were kept on ice and were used to coat the bottom of wells in a 96-well plate (50 µL/well). Matrigel-coated plates were then incubated at 37 °C for 30 min to solidify. HUVECs were seeded on top of Matrigel (5000 cells/well) in EGM-2 with normal glucose levels (NG) or HG + LGF media containing PPN control, PPN-miR-503 inhibitor, or PPN-scramble miRNA. Tubule formation in Matrigel was assessed following 12 h growth at 37 °C and 5% CO_2_ incubation. Images were processed using FIJI Angiogenesis Analyser software (http://image.bio.methods.free.fr/ImageJ/?Angiogenesis-Analyzer-for-ImageJ, accessed on 15 August 2023). Endothelial migration was examined using scratch-wound healing assay. HUVECs were seeded in a 24-well plate at a cell density of 40,000 cells/well in EGM-2 medium for 24 h. Upon reaching a confluent monolayer, a “wound” was created in HUVEC culture by scratching a straight line across the cells using a sterile 200 µL pipette tips. Cells were then washed twice with PBS to remove detached cells. EGM-2 medium or HG + LGF medium was replaced containing PPN treatment accordingly. Cell cultures were incubated at 37 °C and 5% CO_2_. Images for endothelial wound closure were continuously captured every 2 h by Incucyte Live-Cell Analysis system for 24 h. The percentage of migrated area was determined using FIJI Wound Healing Size Tool software (https://github.com/NxSaken/whiplugin, accessed on 15 August 2023).

### 2.6. Hindlimb Ischemia (HLI) 

All animal studies were conducted with ethical approval from Garvan Institute/St. Vincent’s Hospital Animal Ethics Committee (Protocol#20/04). Six-week-old male C57Bl/6J mice were purchased from Australian BioResources. Mice were acclimated for a week prior to the start of any experimental procedures. Diabetes was induced in 7-week-old C57Bl/6J mice by bolus intraperitoneal injection of streptozotocin (STZ, 165 mg/kg in citrate buffer, pH 4.5). Blood glucose levels were monitored with an Accu-Chek glucometer. Mice with fasted blood glucose > 15 mmol/L for 3 consecutive days were classified as diabetic. Surgical procedure of unilateral hindlimb ischemia (HLI) was performed in diabetic mice as previously described [26,27]. In brief, the femoral artery was occluded with double knots using 6-0 silk sutures at the proximal location near the groin and the distal location to the knee. Afterward, the vessel was ligated and excised. A sham procedure was performed on the contralateral limb. Immediately after HLI surgery, PPN controls, PPN-scramble miRNA, or PPN-miR-503 inhibitor (1.75 µg miRNAs/µg PPNs/g of mouse) were injected intramuscularly at the adductor muscles. Blood-perfusion recovery was monitored prior to surgery; immediately post-surgery; and day 3, 5, 7, 10, and 14 post-surgery using the laser Doppler Imaging system (moorLDI2-IR, Moor Instruments, Axminster, UK). Blood flow recovery was expressed as laser Doppler perfusion index (LDPI), a ratio between ischemic and non-ischemic limbs of an individual mouse. 

### 2.7. Immunohistochemistry 

Upon sacrifice, adductor muscles were embedded in Tissue-Tek OCT compound (Finetek, New Taipei City, Taiwan) and snap-frozen in liquid nitrogen and stored at −80 °C. Tissues were cryosectioned at 7 µm thickness and fixed with cold paraformaldehyde (PFA) for 10 min prior to immunohistochemistry staining as described in [26]. In brief, tissue sections were blocked with 5% serum in PBST for 1 h and stained with primary antibodies of anti-laminin (1:250), anti-CD31 conjugated to phycoerythrin (1:250), and anti-α smooth-muscle actin conjugated to fluorescein isothiocyanate (1:1000) overnight at 4 °C. Sections were then washed 3 times with PBST and labelled with secondary antibody and anti-rat IgG conjugated to Alexa Fluor 350 (1:500). Sections were imaged with fluorescence microscopy. The levels of CD31+/myocytes and α-smooth-muscle actin/myocytes were analysed using FIJI software (https://imagej.net/ij/download.html, accessed on 15 August 2023).

### 2.8. Statistical Analysis

Data are expressed as mean ± SEM of at least three independent experiments. Statistical analysis between the two groups was performed using two-tailed, unpaired Student’s *t* test. Statistical analysis of one-way ANOVA followed by Bonferroni or Dunnett’s multiple comparison was used for comparing three or more groups (GraphPad Prism v9.5). *p* < 0.05 was considered statistically significant.

## 3. Results

### 3.1. Characterisation of PPN-miRNA Complex

**Figure 1:**
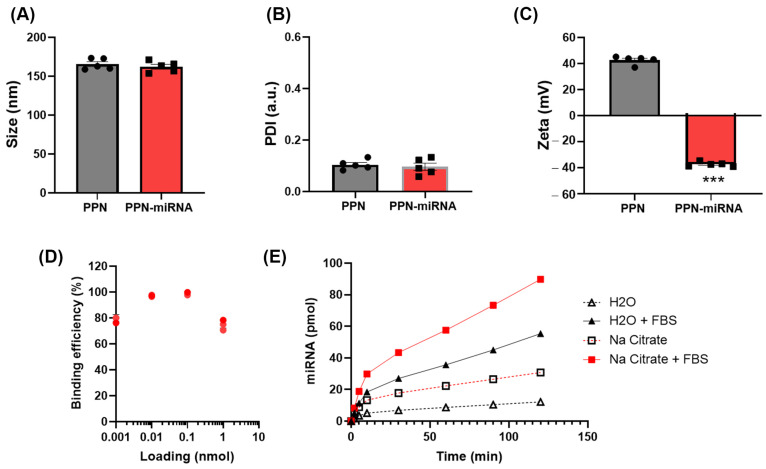
Characterisation of the PPN-miRNA complex. (**A**) Hydrodynamic size, (**B**) polydispersity index (PDI) and (**C**) zeta potential of unfunctionalised and miRNA functionalised PPNs. (**D**) Binding efficiency of PPNs to miRNA. *n* = 5. (**E**) In vitro release profile of miRNA from PPN in nuclease-free water and citrate buffer (pH 7.2) with or without foetal bovine serum FBS. *** *p* < 0.005 vs. PPN. *n* = 3.

To characterise PPN functionalisation with miRNAs, we first examined the hydrodynamic size, polydispersive index (PDI), and zeta potential of the PPN-miRNA complex. The functionalisation of PPN with miRNA did not significantly alter the size (PPN: 162.94 ± 3.653 vs. PPN-miRNA: 153.76 ± 2.392) or PDI (PPN: 0.133 ± 0.047 vs. PPN-miRNA: 0.091 ± 0.041) compared to unfunctionalised PPNs (Figure 1A,B). Meanwhile, the binding of negatively charged miRNA inverted the surface charge of PPNs from +37.10 ± 1.64 mV to −39.04 ± 1.01 mV (Figure 1C). Next, we determined the binding efficiency of PPNs to fluorescently labelled Alexa647-miRNA (Mw = 7998.4 Da) and the release of Alexa647-miRNA in vitro. The binding efficiency of PPNs reached over 95% with 10–100 pmol loading of Alexa647-miRNA. PPN binding efficiency was reduced at higher or lower amounts of miRNA, yet it remained over 75% at all concentrations tested (Figure 1D). On the other hand, a quick release of miRNA from PPNs was observed in sodium citrate buffer at physiological pH 7.2 compared to water. Interestingly, the presence of serum enhanced the release of miRNAs in both citrate buffer and water, as faster release rates were observed in the solution containing FBS (Figure 1E).

### 3.2. Endothelial Uptake and Cytotoxicity of PPN-miRNAs

**Figure 2:**
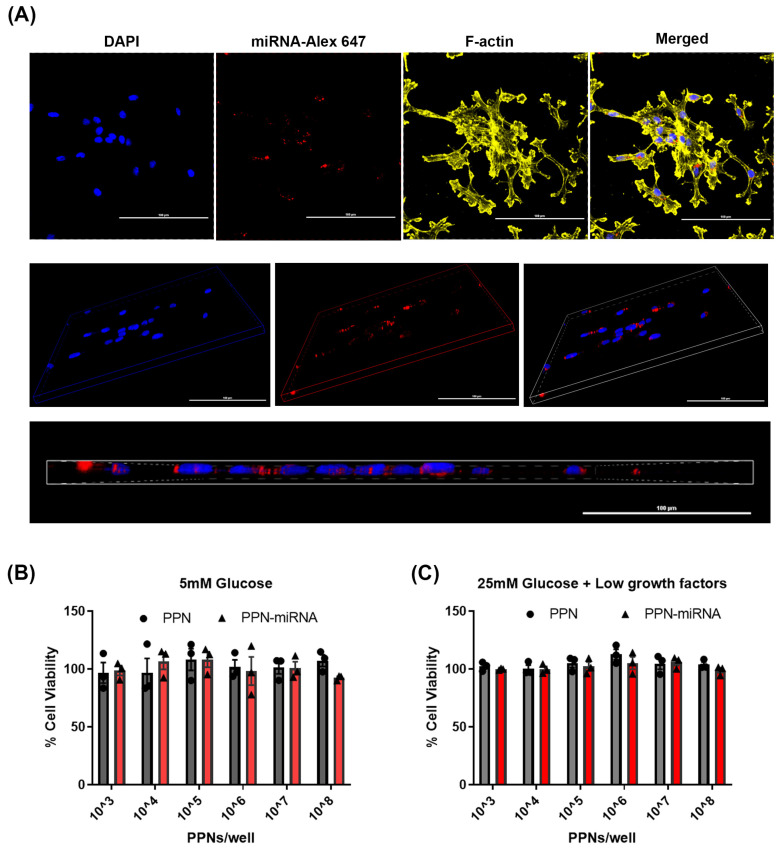
Cellular uptake of PPN-miRNA complex. (**A**) Confocal images of HUVEC uptake of PPN functionalised with Alexa-647-labelled miRNA at 24 h post-exposure. Blue: nucleus staining DAPI; yellow, F-actin; red; PPN-NC5 Alexa647. Scale bar = 100 µm. Fluorescence microscopy images of HUVEC uptake of PPN functionalised with Alex-647-labelled miRNA at 2, 4, and 24 h post-exposure. Cell viability upon delivery of unfunctionalised PPN and functionalised PPN-miRNAs after 3 days treatment. HUVECs were grown in (**B**) 5 mM glucose with fully supplemented growth factors and (**C**) 25 mM glucose and low levels of growth factors. Cell viability was normalised to untreated controls. *n* = 3 independent experiments with technical triplicates.

Endothelial uptake of PPN-miRNA was determined by fluorescence microscopy and FACS analysis. PPN was complexed with Alexa647-miRNA (1.6 µg miRNA/10^9^ PPNs/mL) prior to HUVEC exposure. Cellular uptake of PPN-Alexa647-miRNA was observed as early as 2 h post-exposure with ~ 54% positive cells (Supplementary Figure S2, Figure 2A and Table 1) at this timepoint. FACS analysis showed that the percentage of positive cells increased to over 70% for both 4 h and 24 h exposure of PPN-Alexa647-miRNA (Table 1), indicating an early saturation of PPN-miRNA uptake by endothelial cells. Next, different amounts of miRNAs (25, 50, 100, and 200 pmol) were conjugated to 10^9^ PPNs. Early uptake of PPN-miRNA was independent to the concentrations of fluorescent Alexa647-miRNA with no significant change in the percentage of positive cell populations between groups (Table 2). 

Next, the cytotoxicity of PPN-miRNAs was examined in HUVECs grown under standard laboratory culture conditions with 5 mM glucose and fully supplemented growth factors. HUVECs were treated with a range of PPN concentrations from 10^3^ to 10^8^ particles/0.32 cm^2^ carrying 0.002–200 pmol miRNA (Supplementary Table S1). Compared to untreated HUVECs, both unfunctionalised and miRNA-functionalised PPNs did not significantly affect cell viability (Figure 2B). This indicates that PPNs alone and PPNs complexed with miRNA are well tolerated by endothelial cells under standard culture conditions at these concentrations. We further examined the viability of HUVECs pre-treated with high-glucose and low-growth-factor conditions to mimic diabetes-like conditions in vitro. To mimic ischemia in diabetes, HUVECs were cultured in high levels of glucose (25 mM) without a full supplementation of growth factors (high glucose (HG) + low growth factor (LGF)) [15]. Similar to our results for standard culture conditions, EC viability was not affected by exposure to either PPN or PPN-miRNA, indicating no signs of toxicity, even under disease-mimicking conditions (Figure 2C). 

### 3.3. PPN Delivery of miR-503 Inhibitor Modulates Endothelial Expression of miR-503 and Its Downstream Targets

**Figure 3:**
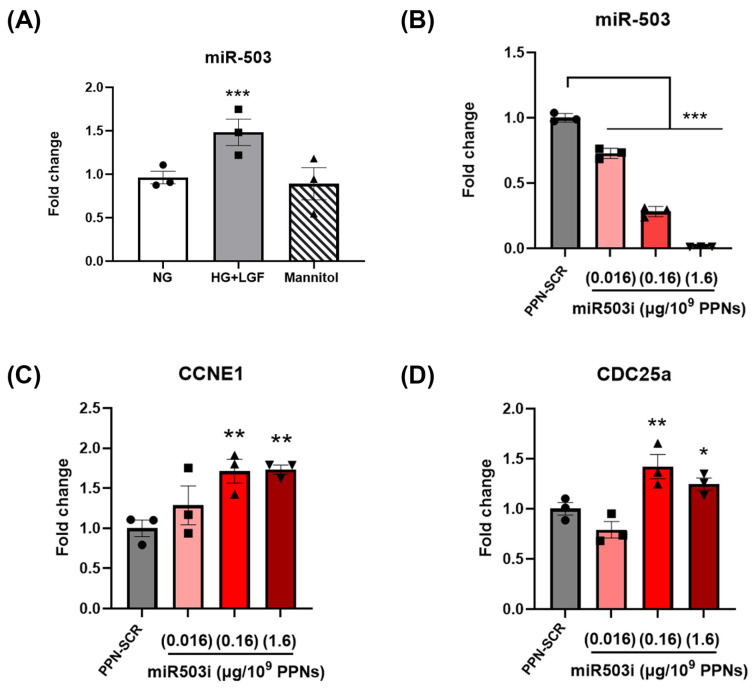
PPN delivery of miR-503 inhibitor modulates miR-503 and its downstream mRNA targets in HG + LGF-grown HUVECs. (**A**) Relative expression of miR-503 in HUVECs grown in culture medium containing 5 mM glucose (NG), 25 mM glucose with low levels of growth factors (HG + LGF), or mannitol. miR-503 expression was normalised to snRU6 expression. Fold changes are expressed as relative NG controls. *** *p* < 0.005 vs. NG-grown cells. (**B**) HUVECs grown in HG + LGF were treated with PPNs complexed with scramble miRNA negative control (SCR) or miRNA inhibitor targeting miR-503 (miR-503i) at concentrations of 0.016 µg miRNA/10^9^ PPNs, 0.16 µg miRNA/10^9^ PPNs, or 1.6 µg miRNA/10^9^ PPNs. Relative mRNA expression of (**C**) *CCNE1* and (**D**) *CDC25a* in HUVECs treated with PPN-miR-503i. Fold changes are expressed relative to PPN-SCR. * *p* < 0.05, ** *p* < 0.01, *** *p* < 0.005 vs. PPN-SCR-treated cells cultured in HG + LGF. *n* = 3 independent experiments with triplicate.

To investigate the effect of PPN delivery of miRNA on endothelial gene expression, we first examined the levels of miR-503, a well-known miRNA biomarker in diabetes. The levels of miR-503 were significantly elevated in diabetic, ischemic-like conditions where ECs were cultured HG + LGF conditions compared to cells grown under normal glucose conditions (5 mM, NG). Mannitol, used as an osmotic control, did not increase miR-503 levels in HUVECs (Figure 3A). The levels of miR-503 in HUVECs treated with PPN controls were comparable to untreated cells in both NG and HG + LGF conditions, indicating that the glucose effect on miR-503 was independent of PPNs (Supplementary Figure S1A). PPN delivery of scrambled miRNA (PPN-scramble miRNA, SCR) did not mitigate miR-503 induction in HG + LGF-grown HUVECs compared to NG-grown ECs (Supplementary Figure S3A). The elevation of miR-503 remained significantly higher than cells grown in NG. In contrast, PPN delivery of the miR-503 inhibitor (PPN-miR-503 inhibitor, miR503i) resulted in a dose-dependent reduction in miR-503 in HG + LGF-grown cells (Figure 3B). 

Next, we determined the effects of PPN delivery of miR-503 inhibitor on the mRNA expression levels of downstream targets of miR-503, CCNE1, and *CDC25a* [15]. HG + LGF conditions suppressed CCNE1 and *CDC25a* expression levels in HUVECs. The mRNA expression of CCNE1 and *CDC25a* was not affected by the presence of PPNs without miRNA (Supplementary Figure S1B,C). Similarly, treatment of PPN-SCR did not restore CCNE1 and *CDC25a* mRNA expression (Supplementary Figure S3). In contrast, PPN delivery of the miR-503 inhibitor (0.16 µg/10^9^ PPNs and 1.6 µg/10^9^ PPNs) significantly increased CCNE1 and *CDC25a* levels compared to PPN-scramble miRNA-treated ECs in HG + LGF (Figure 3C,D). Meanwhile, treatment of the lowest concentration of the PPN-miR-503 inhibitor (0.016 µg/10^9^ PPNs) did not significantly increase CCNE1 and *CDC25a*. Together, these results indicate that PPN delivery of the miR-503 inhibitor suppresses glucose induction of miR-503, subsequently restoring the mRNA expression of its downstream targets, CCNE1 and *CDC25a*.

### 3.4. PPN Delivery of miR-503 Inhibitor Enhances Endothelial Angiogenic Function In Vitro

**Figure 4:**
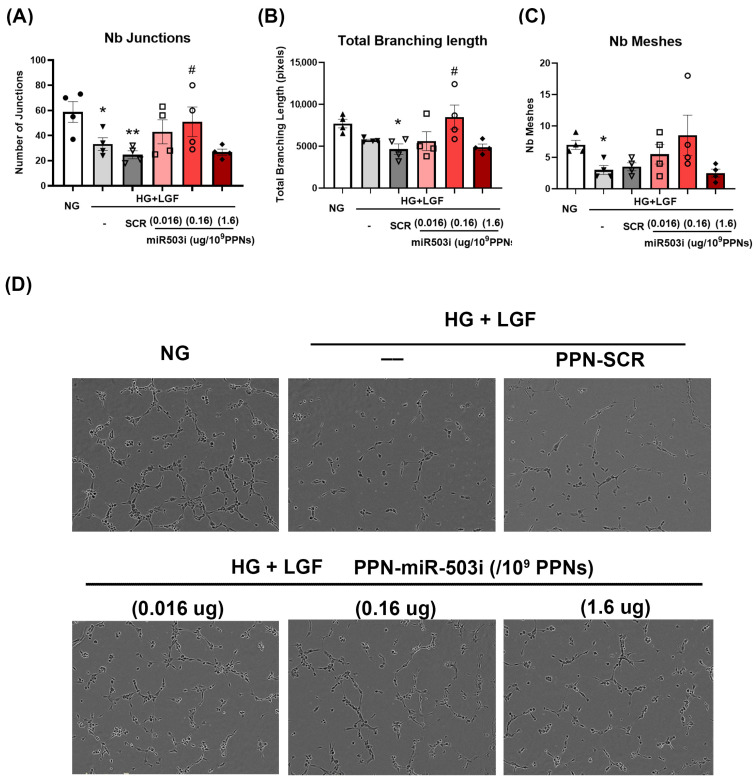
PPN delivery of miR-503 inhibitor enhances endothelial tubulogenesis in HG + LGF. HUVECs were treated with PPN complexed with SCR or miR-503 inhibitor at concentrations of 0.016 µg miRNA/10^9^ PPNs, 0.16 µg miRNA/10^9^ PPNs, or 1.6 µg miRNA/10^9^ PPNs. Tubulogenesis was assessed as tubular network formation in HUVECs cultured on Matrigel and quantified as (**A**) number of junctions, (**B**) total branching length, and (**C**) number of meshes. (**D**) Representative images of tubular network in HUVECs. * *p* < 0.05, ** *p* < 0.01 vs. NG-grown cells; # *p* < 0.05 vs. PPN-SCR treated cells cultured in HG + LGF. *n* = 4.

Given that the expression of CCNE1 and *CDC25a* is key to the survival and angiogenic potential of endothelial cells, we next determined whether PPN delivery of the miR-503 inhibitor rescues EC angiogenic functions in diabetic, ischemic-like conditions. EC angiogenic function was examined by established in vitro tubulogenesis and migration assays. Firstly, tubulogenesis was determined by measuring EC ability to form a tubular network in Matrigel. In comparison to NG-grown HUVECs, EC tubular formation was impaired under HG + LGF conditions as demonstrated by the reduction in numbers of junctions, total branching length, and the numbers of meshes (Figure 4A–D). The reduction in endothelial tubulogenesis in HG + LGF conditions was not mitigated by the presence of PPN alone (Supplementary Figure S4). In contrast, HUVEC treatments with the PPN-miR-503 inhibitor at 0.16 µg miRNA/10^9^ PPNs significantly improved angiogenic function by increasing both the number of junctions and branch length (Figure 4A,B). Interestingly, EC tubular formation was worsened by the higher-dose PPN-miR-503 inhibitor, 1.6 µg miRNA/10^9^ PPNs, which showed no improvement in tubulogenesis compared to PPN-scramble miRNA-treated HUVECs. 

**Figure 5:**
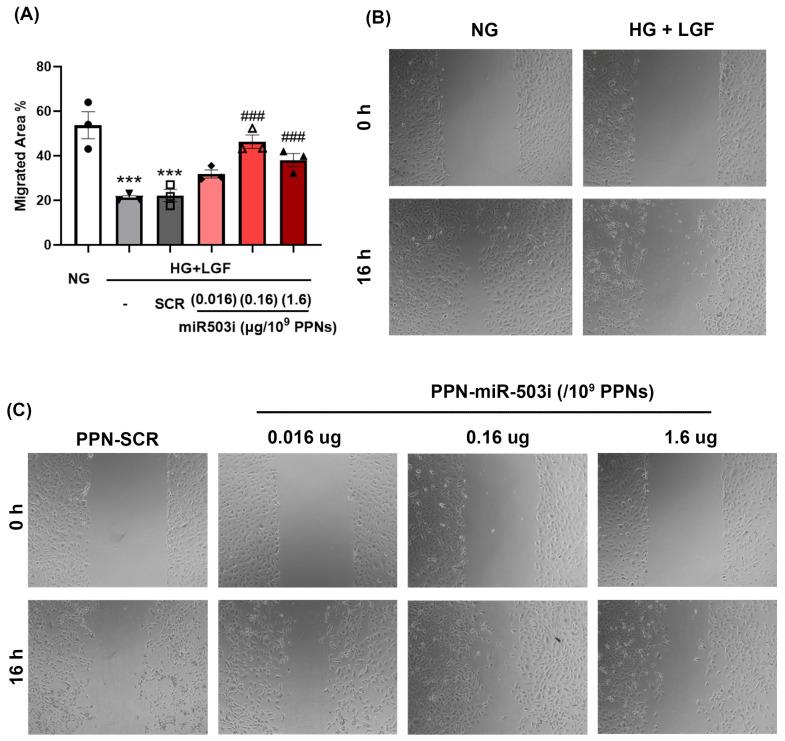
PPN delivery of miR-503 inhibitor promotes endothelial migration in HG + LGF. HUVECs were treated with PPN complexed with SCR or miR-503 inhibitor at concentrations of 0.016 µg miRNA/10^9^ PPNs, 0.16 µg miRNA/10^9^ PPNs, or 1.6 µg miRNA/10^9^ PPNs. Migration was assessed in a scratch-wound assay. (**A**) Endothelial migration is calculated as the percentage of migrated area. (**B**) Representative images of HUVEC migration in NG and HG + LGF conditions at 0 and 16 h. (**C**) Representative images of endothelial migration of HUVECs treated with PPN-scrambled (SCR) or PPN-miR503 inhibitor (miR-503i) in HG + LGF conditions at 0 and 16 h. *** *p* < 0.005 vs. NG-grown cells; ### *p* < 0.005 vs. PPN-SCR-treated cells cultured in HG + LGF.

Secondly, EC angiogenic function was assessed by the ability of cells to migrate through a scratch wound created in a confluent monolayer culture. Similar to our observations in the tubulogenesis assay, EC migratory function was impaired in diabetic, ischemic conditions (Figure 5A,B) and was not restored by the presence of PPN alone (Supplementary Figure S5). PPN delivery of the miR-503 inhibitor (0.16 µg miRNA/10^9^ PPNs) significantly enhanced EC migration by greatly reducing the gap between scratched wounds (Figure 5A,B). While the highest dose of the PPN-miR-503 inhibitor (1.6 µg miRNA/10^9^ PPNs) negatively impacted EC tubular formation, this dosage of PPN-miR-503 inhibitor remained effective in improving EC migration.

### 3.5. PPN Delivery of miR-503 Inhibitor Enhances Blood-Perfusion Recovery Post-Ischemia in Diabetes

**Figure 6:**
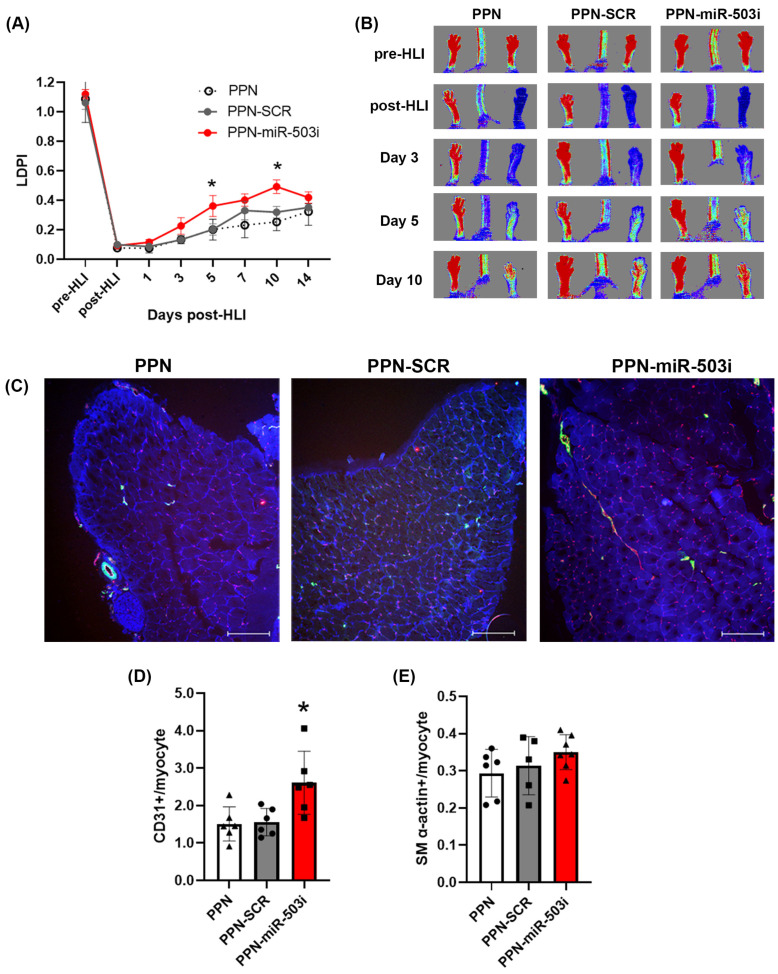
PPN delivery of miR-503 inhibitor improved blood flow recovery in diabetic mice following hindlimb ischemia (HLI). Following HLI surgery, diabetic mice received an intramuscular injection of PPNs, PPN-SCR, or PPN-miR-503 inhibitor. Blood flow recovery was monitored by laser Doppler perfusion imaging for 14 days post-surgery. (**A**) Post-ischemic blood-perfusion recovery was expressed as laser Doppler perfusion index (LPDI), calculated as the ratio between the ischemic limb and the contralateral, non-ischemic limb in diabetic mice. (**B**) Representative colour laser Doppler images. (**C**) Levels of CD31+ per myocytes, (**D**) levels of smooth-muscle α-actin per myocytes. (**E**) Representative images of muscle tissues of diabetic mice post-HLI. Blue, laminin; red, CD31+; green, α smooth-muscle actin. *n* = 6 animals in each group. * *p* < 0.05 vs. PPN controls.

Having demonstrated the potential of the PPN-miR-503 inhibitor in rescuing glucose-induced impairment of EC angiogenic function in vitro, we next investigated the therapeutic potential of PPN delivery of the miR-503 inhibitor in vivo using a mouse hindlimb ischemia (HLI) model. Immediately following HLI, diabetic C57Bl/6J mice received an intramuscular injection of PPN alone or PPN-miRNA treatment with either scrambled miRNA or a functional miR-503 inhibitor. Blood-perfusion recovery was monitored for 14 days post-HLI surgery using a laser Doppler perfusion imager and was expressed as a perfusion index (Supplementary Figure S6). The perfusion index drastically reduced immediately after HLI surgery in all groups, consistent with successful ischemia induction. Mice receiving an intramuscular injection of the PPN-miR-503 inhibitor displayed improved recovery compared to those receiving PPN-scrambled miRNA or PPN-only controls (Figure 6A,B). The perfusion index significantly increased by 44% and 35% for the PPN-miR-503 inhibitor treatment group compared to the scrambled controls on day 5 and day 10 post-HLI, respectively. Histological analysis showed a significant increase in CD31+ per myocytes in the PPN-miR-503 treatment group compared to the scrambled miRNA and PPN-only control groups (PPN-miR-503 inhibitor: 2.61 ± 0.84; PPN-scramble miRNA: 1.56 ± 0.36; PPN control: 1.51 ± 0.46) (Figure 6C). However, there was no significant difference in SMA+ between all groups (PPN-miR-503 inhibitor: 0.35 ± 0.05; PPN-scramble miRNA: 0.31 ± 0.08; PPN control: 0.29 ± 0.06) (Figure 6D). 

## 4. Discussion

In this study, we demonstrated the feasibility of using PPN to deliver functional miRNA both in vitro and in vivo. We observed no cytotoxicity in HUVECs for either PPN alone or PPN-miRNA across a range of doses and showed that PPN-miRNA is rapidly taken up by the endothelial cells. Furthermore, the PPN-delivered miR-503 inhibitor effectively modulates endothelial cell expression of miR-503 and its downstream targets, *CCNE1* and *CDC25a*, thereby enhancing endothelial angiogenesis in diabetes-like conditions as demonstrated by tubulogenesis and migration in vitro. In vivo, we further showed that intramuscular delivery of the PPN-miR-503 inhibitor improves blood flow perfusion in a diabetic mouse model of hindlimb ischemia. 

We developed and characterised a PPN formulation in previous studies [23,25] with physical characteristics that we hypothesised would be favourable for delivering miRNAs in vitro and in vivo. While PPN surface chemistry, charge, and size and can be readily modulated, during the plasma production process, we selected a positively charged (37 mV) ~150 nm particle for this application. The positively charged surface of PPNs enabled robust tethering of negatively charged nucleic acids via electrostatic interaction. Similar to PPN-siRNA complexing defined in a previous study [25], PPN binding to miRNA resulted in an inversion in zeta potential, as the PPN surface charge switched from positive to negative upon successful binding. Despite the change in surface charge, the binding of miRNA did not alter other PPN properties, including size and propensity for aggregation in aqueous solution. Of note, the optimal PPN binding efficiency is determined by the molecular weight of the cargo as it tethers onto the nanoparticle surface [23]. In this study, the optimal binding efficiency of PPN-Alexa647 exhibited a bell shape, where the highest concentration of miRNA may have saturated the optimal molar/surface ratio. 

In addition to its binding efficiency, another advantage of using PPNs to deliver miRNA is attributed to its rapid release of miRNA in physiological-like conditions. Mimicked by citrated buffer at pH 7.2 in vitro, a high degree of miRNA release is achieved within 3 h. In contrast, a gradual release of miRNAs from silicon nanoparticles has been reported to take approximately 8 days [28]. While a gradual release of miRNA has its merits, there are several advantages to achieving rapid release of miRNA from nanoparticles. In addition to enhanced delivery efficiency, rapid release minimises the duration of nanoparticle exposure, which in turn reduces cytotoxicity. It also improves target specificity by reducing the potential of off-target effects. This is particularly important for miRNA-based therapies, where the target miRNA may have some degree of similarity with other miRNAs. 

The use of conventional lipid-based delivery of nucleic acids, such as lipofectamine, is often associated with cytotoxicity. The transfection efficiency comes at the cost of increased cell death. In contrast, PPN delivery of miRNA had no effect on endothelial cell viability, even in diabetes-like conditions in vitro. Considering the therapeutic potential of PPNs, it is critical to evaluate PPN cytotoxicity in both healthy conditions and conditions mimicking disease in target cells. The cellular microenvironment, whether healthy or diseased, can influence nanoparticle uptake and tolerance, including pH, oxygen levels, proliferation states, and the presence of inflammatory or stress responses [29]. For instance, a healthy microenvironment may be less prone to nanoparticle-induced cellular damage due to lower levels of oxidative stress and inflammation. Alternatively, healthy tissues may have more efficient or better controlled clearance mechanisms for nanoparticles, which decrease the risk of over accumulation of nanoparticles, making them less susceptible to the related cytotoxicity. Conversely, a diseased microenvironment with altered metabolic changes or abnormal vascular permeability may lead to increased nanoparticle uptake or accumulation [30]. For instance, it has been demonstrated that cellular uptake of liposome nanoparticles is dependent of glucose transporter GLUT1 [31]. Exploiting this mechanism, nanoparticle glycosylation is one strategy to improve drug delivery in cancerous cells, where glucose transporters are regulated due to increased energy requirement [32,33]. 

In this study, we have chosen a miRNA inhibitor that targets miR-503 in diabetes to investigate the efficiency of PPN delivery. Clinically, miR-503 has been found to be upregulated in diabetic patients with critical limb ischemia who are subjected to lower-limb amputation [15]. In vitro, high-glucose-induced miR-503 upregulation in endothelial cells leads to reduced cell proliferation, migration, and angiogenesis [15]. Local inhibition of miR-503 restores angiogenesis in diabetic mice [15]. Here, we have shown that, using PPN as a delivery vehicle, this miRNA inhibitor suppressed high-glucose-induced upregulation of miR-503 in a dose-dependent manner, which in turn restored the expression of downstream mRNA targets, *CCNE1* and *CDC25a*, to levels comparable to cells grown under normal glucose levels. Interestingly, there is an optimal range of gene expression to achieve the maximal angiogenic function in endothelial cells. While it was noticed that HUVECs tolerated high levels of PPN-miRNAs, the highest concentration of the PPN-miR-503 inhibitor applied to HUVECs did not translate into the best angiogenic performance, as determined by tubulogenesis and migration assays. In Masotti et al. (2016), a miRNA precursor was delivered by polyamine-coated carbon nanotubes to enhance miR-503 expression in endothelial cells [34]. It was shown that delivery of the miR-503 precursor modulated endothelial angiogenic functions and reduced the formation of microvessels in a mouse aortic ring assay ex vivo [34]. Our study not only demonstrates the variety and versatility of nanoparticle technology in delivering miRNAs, but also further showcases the therapeutic potential of PPN delivery of miRNAs in rescuing a diabetic phenotype. We have shown that intramuscular injection of PPN-miRNA improves blood-perfusion recovery in the ischemic hindlimb of diabetic mice. Of note, the diabetic state of the mice was induced by the toxic chemical streptozotocin, such that blood vessel regeneration was severely impaired in this study. Nevertheless, a significant improvement in angiogenesis was achieved by a single administration of PPN-miRNA treatment that targets one specific miRNA, miR-503. 

The use of miRNAs for therapy has distinctive advantages over traditional gene therapy that typically involves genetic materials, such as siRNA, mRNA, or DNA. Firstly, unlike mRNA or siRNA that is specific to one gene, a single miRNA is capable of modulating multiple functionally related downstream mRNA targets, allowing for a wider range of therapeutic targets. Furthermore, miRNAs in the form of mimics and inhibitors can regulate both the expression and repression of multiple genes, while the activity of siRNA and mRNAs is limited to the upregulation or repression of one specific gene. Therefore, miRNA therapy promises a new capability to “correct” complex pathogenic networks. This notion may be an attractive treatment strategy for multifactorial diseases, such as type 2 diabetes or cardiometabolic diseases [35]. In the miR-16 family, where miR-503 belongs, other miRNAs are involved in the development and progression of diabetes [14]. For instance, miR-103 and miR-107 negatively regulate insulin sensitivity and signalling in liver and adipose tissue [36]. MiR-16 and miR-424 regulate hypoxic response and angiogenesis [37]. Importantly, there are many more therapeutic miRNA targets to be investigated in future work. Outside of the miR-16 family, many miRNAs, such as miR-155 [38] or miR-124 [39], are also involved in the pathogenesis of diabetes. Since PPN and miRNAs complex via electrostatic interaction, alteration of miRNA sequences is not expected to influence PPN functionalisation. Additionally, PPN can be used as a vehicle for single or multiple miRNA delivery. In future studies, it may be possible to further enhance the therapeutic potential of PPN-miRNA treatment by targeting multiple miRNAs that facilitate angiogenesis or ischemic response. 

In conclusion, we highlight the therapeutic potential of PPN as a platform for delivering therapeutic miRNA in vitro and in vivo. PPN delivery of miRNA inhibiting miR-503 restored endothelial cell angiogenic function in disease-like conditions through the modulation of target gene expression. Furthermore, in vivo administration of PPN-miRNA improved blood-perfusion recovery in a diabetic mouse model. Together, this study presents a new nanotechnology that facilitates the development of miRNA therapy.

## Figures and Tables

**Table 1:** Percentages of Alexa647-positive HUVECs.

Time (h)	% Alexa647+
2	54.3 ± 4.27
4	70.8 ± 6.29
24	73.8 ± 2.46

**Table 2:** Percentages of Alexa647-positive HUVECs after 2 h treatment.

PPN-miRNA (pmol)	% Alexa647+
25	43.3 ± 3.27
50	52.6 ± 4.29
100	55.7 ± 4.73
200	49.4 ± 5.67

## Data Availability

We included all results in the manuscript and there is no additional data to share.

## Supplementary Materials

The supporting information can be downloaded at: https://www.mdpi.com/article/10.3390/nano13162360/s1.
