# Supplementary material for: Delivery of Therapeutic miRNA via Plasma-Polymerised Nanoparticles Rescues Diabetes-Impaired Endothelial Function
Source: Nanomaterials (Basel). 2023 Aug 18;13(16):2360. doi: 10.3390/nano13162360 (PMC10459051; doi:10.3390/nano13162360)
Supplement: Supplementary file 1 [file nanomaterials-13-02360-s001.zip › Supplementary Figures.pdf]

Supplementary Figures

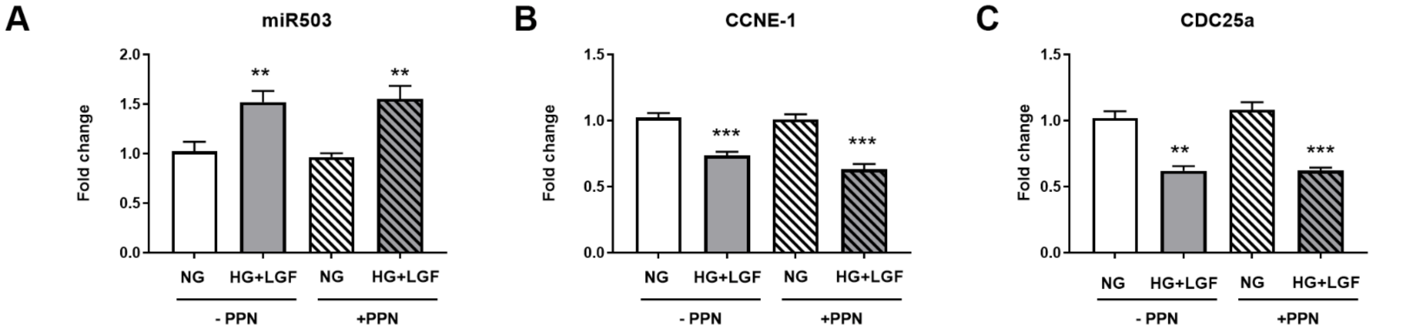

Supplementary Figure S1 Delivery of PPNs alone has no effect on glucose modulation of miR-503 and its downstream mRNA targets, *CCNE1* and *CDC25a*. HUVECs treated with or with PPNs in cultured medium containing 5 mM glucose (NG) or 25 mM glucose with low levels of growth factors (HG + LGF). A) Relative expression of miR-503 in HUVECs. miR-503 ex-pression was normalized to snRU6 expression. Relative mRNA expression of B) *CCNE1* and C) *CDC25a.* mRNA expressions were normalised to housekeeping gene, 18S ribosomal RNA. Data are expressed as fold change relative to NG controls. ** P< 0.01, *** P < 0.005 vs. NG-grown cells

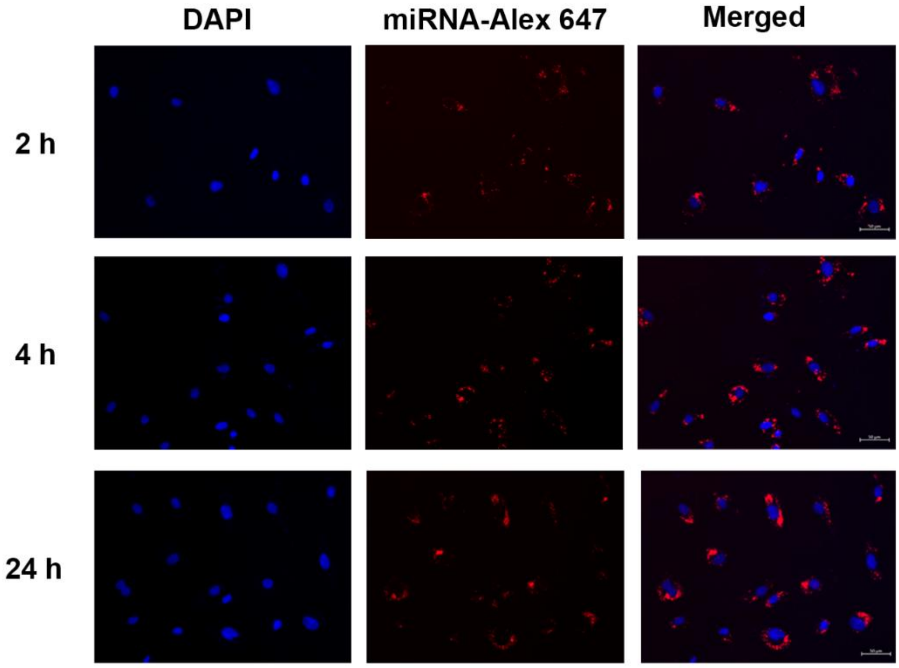

Supplementary Figure S2. Fluorescence microscopy images of HUVEC uptake of PPN functionalized with Alex-647 labelled miRNA at 2, 4 and 24 h post-exposure. PPN was complexed with Alexa647-miRNA (1.6ug miRNA/ 10^9 PPNs/ mL) prior to HUVEC exposure.

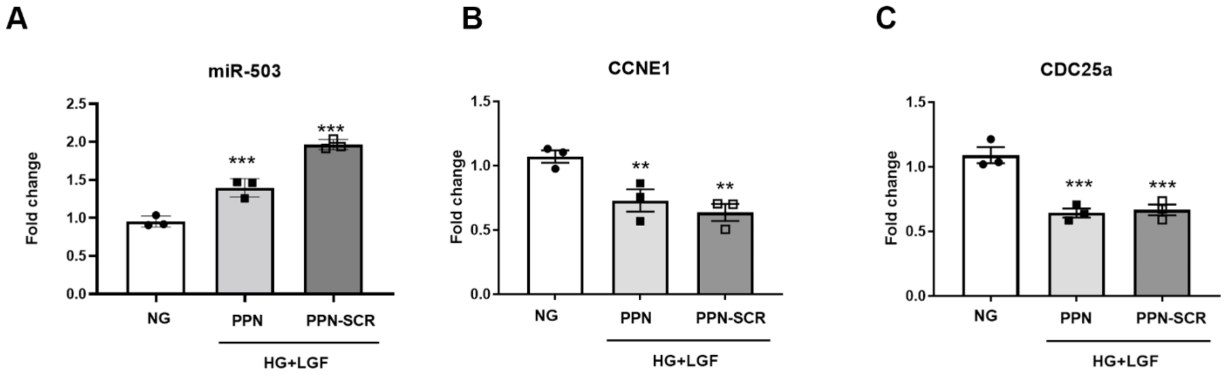

Supplementary Figure S3 Effects of the delivery of PPN alone and PPN-SCR complex in HUVECs. HUVECs treated with or with PPNs in cultured medium containing 5 mM glucose (NG) or 25 mM glucose with low levels of growth factors (HG + LGF). A) Relative expression of miR-503 in HUVECs. miR-503 ex-pression was normalized to snRU6 expression. Relative mRNA expression of B) *CCNE1* and C) *CDC25a*. mRNA expressions were normalised to housekeeping gene, 18S ribosomal RNA. Data are expressed as fold change relative to NG controls. ** P< 0.01, *** P < 0.005 vs. NG-grown cells

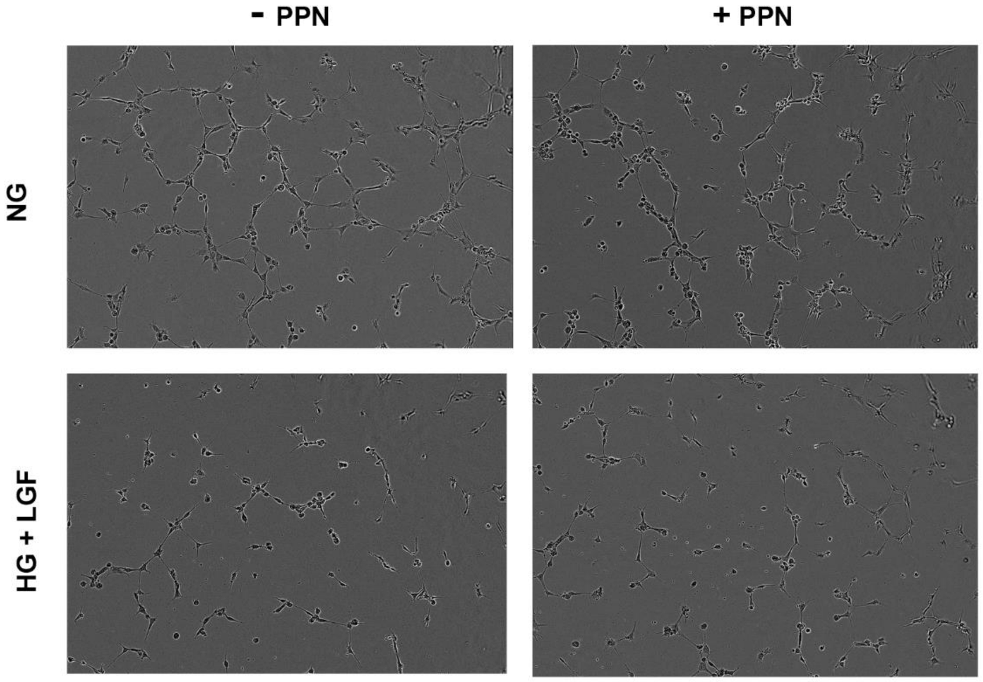

Supplementary Figure S4 Delivery of PPN alone has no effect on tubulogenesis. HUVECs treated with or with PPNs in cultured medium containing 5 mM glucose (NG) or 25 mM glucose with low levels of growth factors (HG + LGF). Tubulogenesis was performed with Matrigel. Representative images of HUVEC tubular formation.

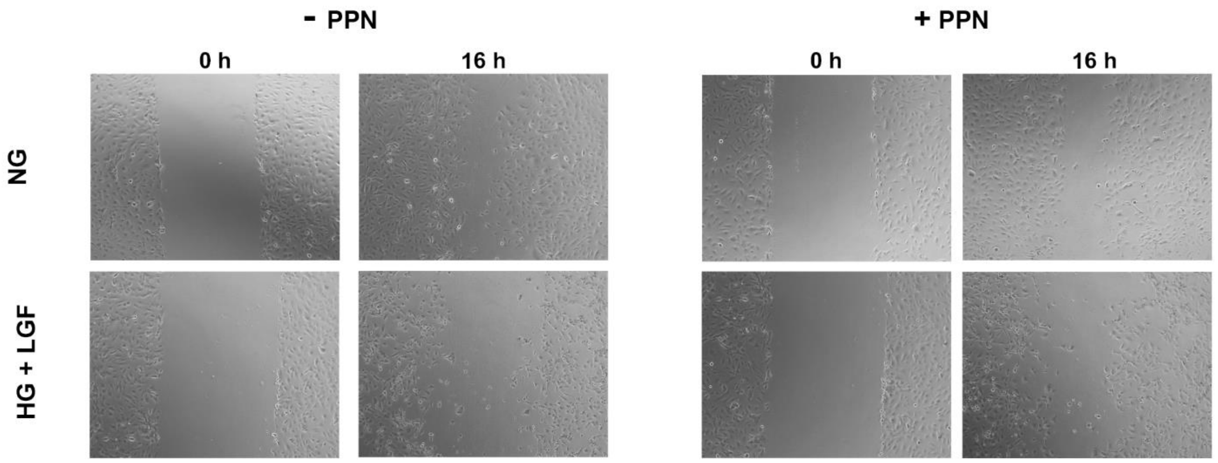

Supplementary Figure S5 Delivery of PPN alone has no effect on endothelial migration. HUVECs were treated with or without PPNs in medium containing 5 mM glucose (NG) or 25 mM glucose with low levels of growth factors (HG + LGF). Migration was assessed in a scratch wound assay. Representative images of HUVECs migration after 16 h.

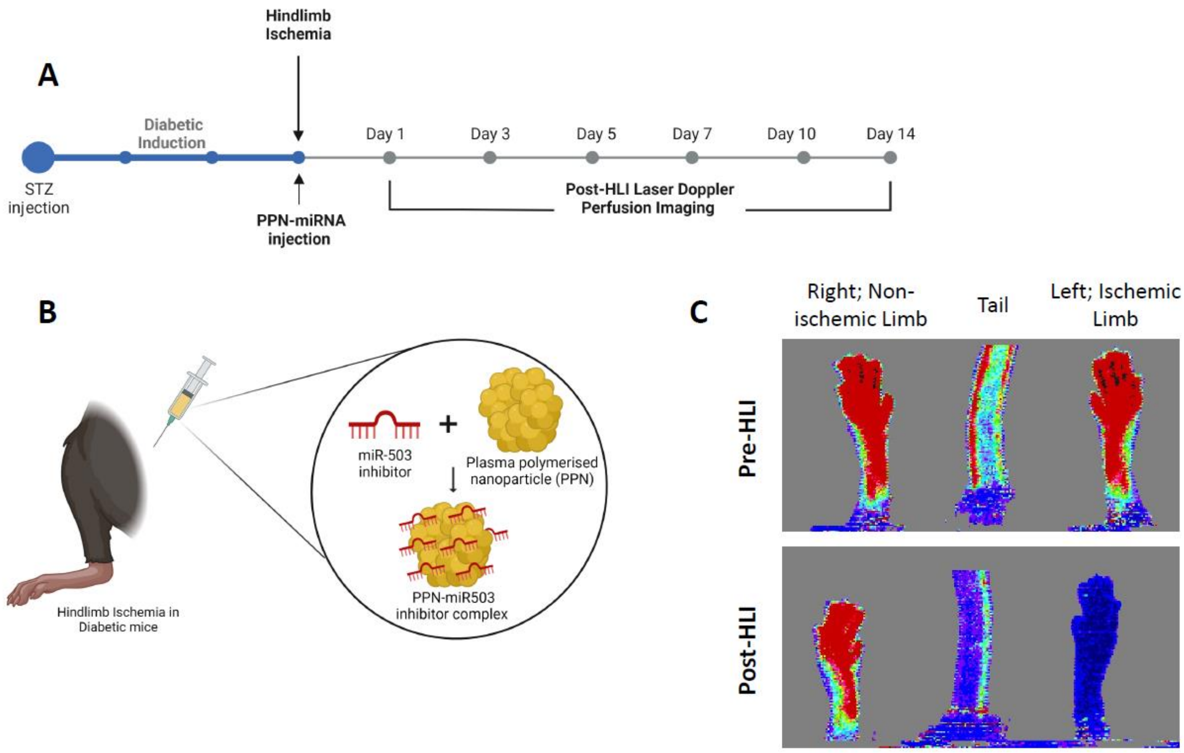

Supplementary Figure S6 Schematic figure of PPN-miRNA in vivo study. A) Experimental timeline of PPN-miRNA treatment on diabetic mice following HLI. B) Schematic diagram of intramuscular injection of PPN-miRNA complex in diabetic mice. C) Schematic view of representative images of laser doppler prefusion imaging.
