# Supplementary material for: Delivery of Therapeutic miRNA via Plasma-Polymerised Nanoparticles Rescues Diabetes-Impaired Endothelial Function
Source: Nanomaterials (Basel). 2023 Aug 18;13(16):2360. doi: 10.3390/nano13162360 (PMC10459051; doi:10.3390/nano13162360)
Supplement: Supplementary file 1 [file nanomaterials-13-02360-s001.zip › Supplementary Tables.pdf]

<u>Supplementary Table</u>

Supplementary Table S1. The concentration of PPN-miRNA complex in HUVECs uptake study

	SCR MW: 5926.9 g/mol; miR-503 inhibitor MW: 5921.85 g/mol)	
**Final PPNs amount**	**miRNA amount**	**miRNA amount (nmol)**
10^8	160 ng	200
10^7	16 ng	20
10^6	1.6 ng	2
10^5	160 pg	0.2
10^4	16 pg	0.02
10^3	1.6 pg	0.002

Supplementary Table S2. The concentrations of PPN-miRNA complex for *in vitro* studies

	(SCR MW: 5926.9 g/mol; miR-503 inhibitor MW: 5921.85 g/mol)	
**Final PPNs amount**	**miRNA amount**	**miRNA amount (nmol)**
10^9	1.6 ug	2
10^9	0.16 ug	0.2
10^9	0.016 ug	0.02

Supplementary Table S3. The concentrations of PPN-miRNA complex for *in vivo* HLI study

**Mouse Weight (g)**	**PPNs (ug)**	**PPN amount**	**miRNA (ug)**	**miRNA (pmol)**
20	20	2.67×10^9	4.27	721
21	21	2.80×10^9	4.49	757
22	22	2.94×10^9	4.70	793
23	23	3.20×10^9	4.91	829
